# Psychosocial group intervention to enhance self-management skills of people with dementia and their caregivers: study protocol for a randomized controlled trial

**DOI:** 10.1186/1745-6215-13-133

**Published:** 2012-08-07

**Authors:** Marja-Liisa Laakkonen, Eeva H Hölttä, Niina Savikko, Timo E Strandberg, Merja Suominen, Kaisu H Pitkälä

**Affiliations:** 1 Department of General Practice, University of Helsinki, P.O. Box 20, Helsinki, 00014, Finland; 2 Helsinki Health Centre, Laakso Hospital, Memory Clinic, Lääkärinkatu 8 F, Helsinki, 00250, Finland; 3 Helsinki Health Centre, Laakso Hospital, Geriatric Psychiatric Clinic, Lääkärinkatu 8 C, Helsinki, 00250, Finland; 4 Department of General Internal Medicine and Geriatrics, Helsinki University Central Hospital, P.O. Box 340, Helsinki, HUS, 00029, Finland; 5 Institute of Health Sciences/Geriatrics, University of Oulu, and Oulu University Hospital, Unit of General Practice, Oulu, Finland; 6 Society for Memory Disorders Expertise in Finland, Fredriksberginkatu 2, Helsinki, 00240, Finland; 7 Unit of General Practice, Helsinki University Central Hospital, P.O. Box 340, Helsinki, HUS, 00029, Finland

**Keywords:** Self-management, Dementia, Caregiving, Quality of life, Empowerment, Self-efficacy, Group rehabilitation

## Abstract

**Background:**

After diagnosis of a dementing illness, patients and their spouses have many concerns related to the disease and their future. This often leads to poor psychological well-being and reduced health-related quality of life (HRQoL) of the family. Support for self-management skills has been proven to be an effective method to improve prognosis of asthma, heart failure and osteoarthritis. However, self-management interventions have not been studied in dementia. Therefore, our aim was to examine, in an objective-oriented group intervention, the efficacy of self-management support program (SMP) on the HRQoL of dementia patients and their spousal caregivers as well as on the sense of competence and psychological well-being of caregivers.

**Methods:**

During the years 2011 to 12, 160 dementia patients and their spouses will be recruited from memory clinics and randomized into two arms: 80 patients for group-based SMP sessions including topics selected by the participants, 80 patients will serve as controls in usual community care. Sessions may include topics on dementia, community services, active lifestyle and prevention for cognitive decline, spousal relationship, future planning and emotional well-being. The patients and spouses will have their separate group sessions (ten participants per group) once a week for eight weeks. Main outcome measures will be patients’ HRQoL (15D) and spousal caregivers’ HRQoL (RAND-36), and sense of competence (SCQ). Secondary measures will be caregivers’ psychological well-being (GHQ-12) and coping resources, patients’ depression, cognition and signs of frailty. Data concerning admissions to institutional care and the use and costs of health and social services will be collected during a two-year follow-up.

**Discussion:**

This is a ‘proof-of-concept’ study to explore the efficacy of group support for self-management skills among dementia families. It will also provide data on cost-effectiveness of the intervention.

**Trial registration:**

ACTRN12611001173987

## Background

Cognitive disorders, such as Alzheimer disease, gradually leading to the dementia syndrome are the most important chronic illness group leading to increased need for assistance, disability and institutional care among older people [1]. Dementia also augments stress and burden, and reduces quality of life (QoL) among caregivers [2]. Usually, dementia patients wish to live at home as far as possible and the spousal caregivers are in the key role in enabling it. Therefore, there is an urgent need for developing supportive methods for coping, as well as effective psychosocial rehabilitation for patients with dementia and their caregivers alike.

Today, there is growing evidence that the most effective interventions for dementia are psychosocial interventions focusing on the needs of both patient and caregiver. Using these interventions, dementia families are provided with means to adapt and cope with dementia [3]. Psychosocial interventions coach caregivers to help patients to use their remaining capacities to participate. Effective psychosocial interventions are built on co-operation between professionals and dementia families [4]. By supporting mastery, self-efficacy and problem-solving skills of caregivers, these interventions aim to empower dementia families to cope with their everyday life, promote dignity and autonomy [5]. Also, the concept of reciprocity in giving and receiving support has been highlighted [6]. Many psychosocial studies demonstrate that individuals can be supported to maintain their skills and build resilience [4,7,8].

Many health-promoting programs have used self-management methods and they have been shown to be effective in improving prognosis of chronic diseases including asthma,diabetes, and heart failure [9,10]. Self-management can be defined as a person’s ability to organize his/her life under the influence of a chronic disease, to engage in activities and protect and promote health through knowledge. Self-management programs (SMP) combine biological, psychological and social intervention techniques to maximize functioning [9,11]. In SMP, professionals support patients with a patient-centered approach to manage their care [12,13]. This includes support of patient’s autonomy, decision making, problem-solving skills and responsibility [5,12,14]. It places patient’s values, needs and priorities in the center of health care [15]. In SMP, health professionals act as coaches or collaborators - partners - rather than as experts, and they respect the patients’ everyday know-how. Central concepts are patient’s empowerment, support of self-efficacy and mastery [12].

Self-management has been less studied in the context of dementia although many trials examining psychosocial support have included features of self-management, elements like family-centered services, respecting autonomy of caregiving families, empowering caregivers and developing cooperation [4,8,16]. In a Finnish SMP, the dementia patients and their spousal caregivers are seen as experts of their own life and consequently expected to take an active role regarding their health and illness [4,5]. SMP differs from traditional knowledge-based education; it includes dimensions such as helping patients and their caregivers to identify the problems, developing their own problem-solving skills and improving their self-efficacy to master their everyday life. Professionals’ optimistic orientation is the basement in SMP [4,5].

Earlier, the diagnosis of dementing illness was often associated with hopelessness and stigma. Nowadays, the dementia is settled earlier during the course of the disease. Thus, patients are cognitively more capable to cope with the disease. Professionals are required to give more emotional support, and provide patients also with practical skills and techniques. Often the patients have few opportunities to talk about their worries after the disclosure of diagnosis [17]. SMP intervention should be centered on patient and carers alike [18].

A recent paper described an English model of SMP for dementia patients and their caregivers [19]. The model included previous elements of psychosocial interventions and health promotion. SMP supported families to be physically, mentally and socially active, and it promoted knowledge for understanding and managing with dementia [19]. Facilitators guided the SMP groups of patients and caregivers to select the most relevant topics and assisted participants to explore them [19].

In the present study, we examine the self-management program (SMP) method among couples with dementia in a clinical trial. It is based on evidence-based knowledge of dementia care and healthy well-being. The aim is to examine the effectiveness of SMP in an objective-oriented group intervention, including empowerment of participants, support of their mastery and self-efficacy. The primary aim is to explore the effects of intervention on dementia patients’ QoL and on spousal caregivers’ QoL, sense of competence and psychological well-being. The secondary aims are to assess the effects of group intervention on patients’ depression, cognition and signs of frailty and on caregivers’ depression and self-efficacy. We also study the effects of intervention on the use and costs of health care services, dementia patients' time to be at home and mortality.

## Methods

### General design

This is a randomized, controlled, prospective intervention trial to examine the effectiveness of the SMP using objective-orientated group intervention where participants’ empowerment, mastery and self-efficacy are supported.

The intervention period lasts for two and a half months, and is compared with usual care. The study protocol and its amendments were approved by the Ethics Committee of Helsinki University Central Hospital (decision number 340/13/01/2010, 3/2011 and 8/2011) and by the chief executive of the Health Centre of the City of Helsinki. Informed consent was obtained from each patient and/or their spousal caregiver before any study procedures. An executive committee (MLL, KHP) is responsible for the planning, conducting and monitoring of the study.

### Participants

The recruiting has started in 2011 from memory clinics and from private neurologists’ or geriatricians’ consultation offices in the greater Helsinki area (Helsinki, Espoo, Vantaa). Patients who have received dementia diagnosis after full diagnostic procedures recommended by the Finnish national guidelines [20] (fulfilling criteria for probable Alzheimer disease diagnosis according to NINCDS-ADRDA Alzheimer’s criteria) and who live at home with their spouse are eligible to be recruited to the study.

Other inclusion criteria are:

Finnish speaking

living in the greater Helsinki area (Helsinki, Espoo, Vantaa)

not in the terminal phase of illness (estimated prognosis > one year)

is able to move independently (with or without devise)

no severe hearing loss that impedes participation in the group

the ability to concentrate in a group situation for two hours without a spousal caregiver

Those couples fulfilling the inclusion criteria are invited to take part in this intervention study. The information sheet about the study is given to the couple in the memory clinic or when visiting a private specialist after diagnostic procedures. A study nurse interviews the couple by phone to makes sure that they fulfill the inclusion criteria. After this, oral information about the study is given, and an appointment is made for the first visit. At the beginning of the first visit, the couples are given written and oral information of the study and both are asked to sign an informed consent. In the case of the dementia patient’s poor capability of judgment, the spousal caregiver gives a proxy’s consent for both spouses.

### Study procedures

The baseline visit lasts about one hour and includes interviews of both spouses and collection of demographic data, diagnoses, current medications, and baseline use of health and social services. Diagnoses and medications are confirmed from medical records provided by the couples. Charlson’s comorbidity index is calculated to assess the severity and prognostic value of the participants' disease burden [21].

Health-related quality of life (HRQoL) of the patients is assessed by using the 15D instrument [22]. Dementia patients are also assessed with the Clinical Dementia Rating Scale (CDR) [23], the Mini Mental State Examination (MMSE) [24], verbal fluency [25], and the clock drawing test [26]. Other measurements include activities of daily living (ADL) [27], instrumental activities of daily living (IADL) [28], the Mini Nutritional Assessment (MNA) [29], Cornell depression test [30], Illness Cognition Questionnaire [31], frailty criteria [32], and the Psychosocial Well-being Scale [33]. The presence of advance directives, such as a living will, are inquired about. Clinical measurements include height, weight, blood pressure and hand grip strength.

The spousal caregivers are asked questions concerning the impact of their spouse’s dementia diagnosis and they are assessed by the RAND-36 HRQoL instrument [34,35], 12-item General Health Questionnaire (GHQ-12) [36], the Center for Epidemiologic Studies Depression Scale (CES-D) [37], Pearlin Mastery Scale for their personal coping resources [38], and the Sense of Competence Questionnaire (SCQ) [39]. Their weight and blood pressure is also assessed on each visit.

The couples are randomized in clusters of 20. After acquiring informed consent to the study and assessing 20 couples with eligibility criteria, the randomization is performed by telephone to a randomization center. Couples are randomly allocated by means of computer-generated random numbers. Every randomization result will appear in the program after the participants name has been written and the person executing the randomization has confirmed the process with her initials. This ensures that neither the study nurse, nor person doing the randomization can influence the result. Half of the participants are randomized to the rehabilitation group (intervention group) and the other half receives usual care (control group).

**Figure 1:**
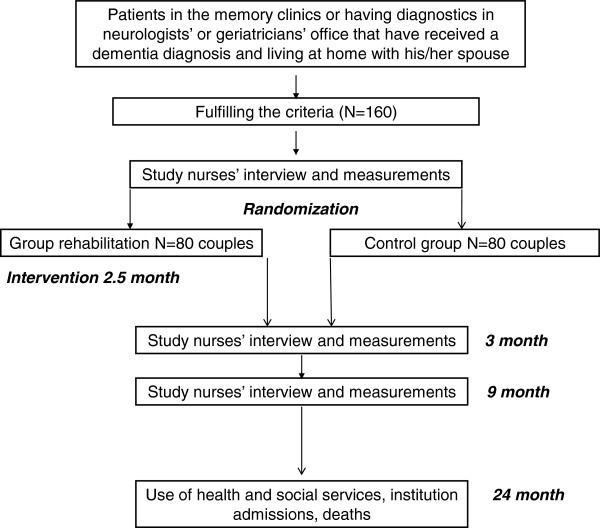
The flow chart of the study.

We aim for a total of 160 couples to be randomized in two groups: group rehabilitation as the intervention and normal care as the control group. Participating couples are assessed by two study nurses three times over nine months: at baseline, three, and nine months. Use of health services, institutionalizations and mortality of both patients and caregivers will be retrieved from the central registers until 24 months from the baseline measurements. The flow chart of the study is presented in Figure 1, and the study assessment procedures are described in Table 1.

**Table 1:** Study assessments, procedures and timetable

**Assesment**^**1**^	**Telephone interview**	**Baseline visit**	**3-month visit**	**9-month visit**	**At 24 months**
Patient					
Inclusion criteria	X				
Demographics, diagnoses, drugs, background information		X	X	X	
CDR, MMSE, verbal fluency, clock drawing test		X	X	X	
Grip strength, blood pressure and pulse		X	X	X	
MNA		X	X	X	
Weight, BMI		X	X	X	
ADL, IADL		X			
Cornell Depression Scale		X	X	X	
Illness Cognition Questionnaire		X	X	X	
15D QoL		X	X	X	
Use of health and social services, mortality			X	X	X
Psychosocial well-being scale		X	X	X	
Spousal caregiver	X	X	X	X	
Demographics, diagnoses, drugs, questions related to coping with dementia		X	X	X	
Psychosocial well-being scale		X	X	X	
Grip strength, blood pressure and pulse		X	X	X	
Weight, BMI		X	X	X	
Sense of competence, SCQ		X	X	X	
CES-D		X	X	X	
12-GHQ		X	X	X	
Pearlin Mastery Scale		X	X	X	
Rand-36 QoL		X	X	X	
Use of health and social services, mortality			X	X	X

^1^*CDR* Clinical Dementia Rating Scale [23] (0.5 suggests possible dementia, 1 mild, 2 moderate, and 3 severe dementia), *MMSE* Mini Mental State Examination [24]. *MNA* Mini Nutritional Assessment [29] (<24 suggests risk for malnutrition or malnourishment); *BMI* body mass index; *ADL* activities of daily life [27]; *IADL* instrumental activities of daily living [28]; Cornell Depression Test [30]; Illness Cognition Questionnaire [31]; 15D QoL, measure of health-related quality of life [22]; Psychosocial Well-being Scale [33]; *SCQ* Sense of Competence Questionnaire - [39]; *CES-D* The Center for Epidemiologic Studies Depression Scale [37]; 12-*GHQ*, 12-item General Health Questionnaire. Pearlin Mastery Scale [38];Rand-36 QoL [34,35].

### Intervention

Group facilitators visit the couples prior to the group rehabilitation in order to get acquainted with them, and to encourage them to express their wishes and preferences for topics for the group sessions. The groups of 10 participants meet once a week for eight weeks. Patients and spousal caregivers will meet their peers in their own groups concurrently. The group meetings last for four hours including lunch, taxi transportation will be provided. Both groups are facilitated by two trained professionals, and the content of the sessions varies according to participants’ preferences. They may include, for example, topics on dementia and prevention of further cognitive decline, active lifestyle and emotional well-being, spousal relationship, future planning and community services. Participants are also advised to collect a folder of important information and tips to overcome difficulties with dementia. Couples are encouraged to define their individual goals for dementia care. Dementia patients having Cornell scale [30] points > 10, and caregivers having CES-D [37] points > 20, or suffering from significant burden are offered a geropsychiatric consultation (EH).

Groups work on the basis of the psychosocial group rehabilitation model described earlier [33,40] and on self-management supporting principles [5,19]. They are based on a constructive learning theory and a reflective learning model, in which self-management skills are built little by little during the intervention. Different kinds of active learning methods are used in the groups, like working in pairs and brainstorming sessions. Group intervention is goal-oriented and it takes advantage of group dynamics and peer support. The aim is to enhance the active agency of older people, to empower them, and to increase their feelings of self-efficacy and mastery. The intervention is tailored according to the wishes of group participants. It, for example, provides participants with knowledge (about, for example, dementia, active lifestyle, nutrition) and skills (for example problem solving and control of everyday family life, goal setting) and so strengthens their coping skills.

Facilitators receive training for group facilitation (the ‘Circle of Friends’ model) [41] and they receive tutoring through the group facilitating process. Facilitators write diaries of the group meetings that enable the researchers to follow and tutor the group activities. In addition, some group meetings are observed by researchers and facilitators receive constructive feedback.

### Outcome measurements

Primary outcome measures are changes in patients’ HRQoL assessed by 15D [22] and in spousal caregivers’ HRQoL assessed by RAND-36 [35], sense of competence assessed by SCQ [39]. Secondary outcome measures are patients’ time spent at home and changes in patients’ depression (Cornell) [30], and feelings of acceptance and helplessness assessed using subscales of the Illness Cognition Questionnaire [31] and cognition (verbal fluency, the clock drawing test (CDT)) [25,26]. Secondary outcome measures for caregivers are psychological well-being assessed by 12-GHQ [36] and personal coping resources by the Pearlin Mastery Scale [38] and also changes in depression will be measured by CES-D [37]. Weight, blood pressure and hand grip are measured as secondary outcomes in the couples. Total mortality, use and costs of health care and social services of both spouses as well as cost-effectiveness of the intervention are measured up to 24 months from the beginning of the baseline measurements.

The study is made in co-operation with the Health Care Centre and Social Services Department of the City of Helsinki, the University of Helsinki, the Central Union for the Welfare of the Aged, and the Rehabilitation Hospital of Oulunkylä.

### Statistical analyses

Sample size was calculated based on the 15D measure [22]. With an estimated standard deviation of 0.10, and type I error of 5%, and 80% power, 62 patients would be needed in each group to show a 10-point difference between groups. We estimated that 20% will drop out so we decided on 160 patients as a sample size. Data will be analyzed on an intention-to-treat basis. In baseline findings, the continuous variables and descriptive values will be expressed by means with standard deviations (SD) and medians with range. For the variables with a normal (Gaussian) distribution, statistical comparisons between the groups will be made by using an analysis of variance. If the variables have a non-normal distribution or ordinal level, statistical comparison between groups will be made using the Mann–Whitney U test. Measures with a discrete distribution will be expressed as percentages (%) and analyzed by chi-square or Fischer's exact test when appropriate. Imputation method of ‘the last observation carried forward’ (LOCF) and ‘worst-rank score’ principle will be used.

Since the distributions of health care costs are highly skewed, the differences between means and confidence intervals are estimated using the bootstrap method (bias corrected and accelerated bootstrapping).

## Discussion

To our knowledge, this trial is the first one to test a support program of self-management among dementia families. We investigate in our trial the effects of an eight-week psychosocial group intervention to support the self-management skills of home-dwelling patients with dementia and their spousal caregivers. SMP intervention is compared with usual care. This intervention is provided for four hours per week in a day center. The group intervention is based on peer support, use of group dynamics and empowerment of participants to take active agency in their life. The SMP will encourage participants to identify their strengths and to enhance their problem-solving skills. This study also provides data on cost-effectiveness of the intervention.

There are several strengths in our study. First, all participants have an established diagnosis of dementia because they are recruited from memory clinics or after diagnostic procedures supervised by neurologists or geriatricians with CT or MRI scans, cognitive and laboratory tests. This is the diagnostic procedure recommended by national guidelines 2010 [20] and the Social Insurance Institution of Finland which controls the drug imbursements including all Alzheimer drugs. The national diagnostic scheme [20] applied to practically all new Alzheimer cases ensures that diagnosis is in line with the Diagnostic and Statistical Manual of Mental Disorders, Fourth Edition (DSM-IV) and the National Institute of Neurology and Communicative Disorders and Stroke-Alzheimer’s Disease and Related Disorders Association (NINCDS-ADRDA) Alzheimer’s criteria. Second, all group leaders are trained very carefully and intensively. We tutor their work to ensure the uniform quality of the intervention, and also support the commitment of participants to the groups.

There are also challenges in this study. The population is old and frail with comorbidities and, therefore, prone to competing causes of complications and death. The intervention is delivered in a multifactorial and flexible format, so it is difficult to determine which aspects of intervention are efficacious. It is possible that there is heterogeneity in the continuation of meetings with group members after the eight weeks of organized meetings, which, with dropping out, may contribute to insufficient difference between the intervention and normal care groups. Contamination of the control group presumably is not a problem, because psychosocial group rehabilitation is rarely available for dementia patients and their caregivers in Finland. It is also challenging to know if the selected scales are suitable and sensitive to measure changes in self-efficacy, mastery, competence and especially HRQoL and depression in this heterogeneous group consisting of various stages of dementia.

The group dynamics and atmosphere are unique in each group. It may be possible that the dementia patient is happy in their group, but the caregiver feels burden or bored in their own group and, therefore, wishes to drop out of the intervention. It is important that the group leaders communicate with each other to know how the ‘other spouse’ works in the other group.

The trial follows common ethical principles in randomized trials. Participants are not exposed to risks, they receive verbal and written information before any study procedures, they participate voluntarily, and they may withdraw at any phase of the study. Individuals with dementia write an informed consent when they are capable of doing so; otherwise the spousal caregiver writes it on behalf of both of them. Control group members receive normal care offered by community. For the rehabilitation and study nurse visits, a risk/liability insurance is taken for participating dyads.

## Trial status

Patient recruitment is open.

## Abbreviations

ADL: Activities of daily life; BMI: Body mass index; CDR: Clinical dementia rating scale; CES-D: The center for epidemiologic studies depression scale; GHQ-12: 12-item general health questionnaire; HRQoL: Health-related quality of life; IADL: Instrumental activities of daily living; MMSE: Mini mental state examination; MNA: Mini nutritional assessment; NINCDS-ADRDA: National institute of neurology and communicative disorders and stroke-alzheimer’s disease and related disorders association; RAND-36: Measure of health-related quality of life; SCQ: The sense of competence questionnaire; SMP: Self-management program; QoL: Quality of life.

## Competing interests

Dr Laakkonen has been working for the Memory Clinic in the Helsinki Health Centre. Dr Eeva Hölttä has been working for the Geriatric Psychiatric Clinic in the Helsinki Health Centre. All the other authors have no competing interests.

## Authors’ contributions

Conception and design (MLL, KHP, TES, EHH, NS, MS) acquisition of data, or analysis and interpretation of data (MLL, KHP, TES) drafting or critically revising the manuscript for important intellectual content (MLL, KHP, TES, NS, EHH, MS) approval of the final manuscript (MLL, KHP,TES, NS, EH, MS). MLL and KHP had full access to all of the data in the study and take responsibility for the integrity of the data and the accuracy of the data analysis. MLL is the guarantor. All authors read and approved the final manuscript.
